# Efficacy of Problem Based Learning approach for teaching Evidence Based Practice in midwifery and nursing education: a systematic review

**DOI:** 10.1186/s12912-025-04101-w

**Published:** 2025-11-19

**Authors:** Grace Komuhangi, Florian Neuhann, Valerie R. Louis, Moses Ocan, Alison Annet Kinengyere, Jürgen Wacker

**Affiliations:** 1 Heidelberg Institute of Global Health, University Hospital and Medical Faculty, Heidelberg University, Im Neuenheimer Feld 130.3, 69120 Heidelberg, Germany; 2 School of Medicine and Allied Health Sciences, Levy Mwanawasa Medical University, Lusaka, Zambia; 3 Department of Pharmacology & Therapeutics, College of Health Sciences, Makerere University, Kampala, Uganda; 4 Albert Cook Library, College of Health Sciences, Makerere University, Kampala, Uganda; 5 Department of Obstetrics and Gynecology, Faculty of Medicine, Heidelberg University, Im Neuenheimer Feld 440, 69120 Heidelberg, Germany

**Keywords:** Problem-Based Learning, Evidence-Based Practice, Nursing education, Midwifery education, Systematic review, Healthcare education

## Abstract

**Background:**

Problem-Based Learning (PBL) has emerged as a promising strategy for teaching Evidence-Based Practice (EBP) to nursing and midwifery professionals. However, its effectiveness remains insufficiently synthesized, with few studies directly addressing its impact on EBP competencies.

**Objective:**

To systematically review and synthesize evidence on the efficacy of Problem-Based Learning as a teaching approach for developing Evidence-Based Practice competencies among nursing and midwifery students and professionals.

**Methods:**

This systematic review with narrative synthesis was conducted in accordance with the Cochrane Handbook and reported following PRISMA 2020 guidelines. The protocol was registered in PROSPERO (CRD42023390989). A comprehensive search of MEDLINE, CINAHL, PubMed, EMBASE, Web of Science, ERIC, PsycINFO, and Cochrane CENTRAL was performed from inception through August 2025. Two reviewers independently screened and appraised studies using JBI-SUMARI tools and ROBINS-I.

**Results:**

Eighteen studies met inclusion criteria, but only three (16.7%) specifically examined PBL for EBP education among nursing students in Iran, Japan, and South Korea. These consistently demonstrated significant improvements in knowledge, attitudes, critical thinking, and clinical decision-making compared with traditional methods, with all rated at low risk of bias. Broader EBP education studies reported highly variable implementation rates (13–100%), with higher educational levels linked to greater uptake of evidence-based behaviours. Across studies, factors supporting success included adequate time, access to technological resources, faculty preparation, and organizational support. Common barriers were limited infrastructure, faculty workload, and students’ initial resistance to active learning.

**Conclusions:**

Preliminary evidence suggests PBL enhances EBP-related competencies, but the current evidence base is small, heterogeneous, and lacks midwifery-specific studies. Rigorous comparative research using standardized outcomes and evaluating long-term practice impact is needed to establish optimal strategies for integrating PBL into nursing and midwifery education.

**Supplementary information:**

The online version contains supplementary material available at 10.1186/s12912-025-04101-w.

## Background

The evolution of nursing and midwifery education from apprenticeship models to academic institutions represents a crucial transformation in healthcare professional development. This shift, occurring predominantly in the latter half of the 20th century, fundamentally changed how Evidence-Based Practice (EBP) is integrated into educational curricula [1].

Evidence-Based Practice represents a systematic approach to clinical decision-making that integrates the best available research evidence with clinical expertise and patient values [2]. The integration of EBP into nursing and midwifery curricula demonstrates the profession’s commitment to evidence-based healthcare delivery while acknowledging the ongoing need to bridge theoretical knowledge with clinical practice [3].

However, significant challenges exist in teaching and learning EBP methodologies. These challenges include difficulties in accessing and appraising research evidence, limited time for evidence searching, insufficient critical appraisal skills, and resistance to changing established practices [4, 5]. Healthcare educators face additional barriers including lack of resources, inadequate technological infrastructure, and limited expertise in evidence-based teaching methods [6].

Problem-Based Learning (PBL) has emerged as a potential solution to these educational challenges. Problem-based learning is an instructional method in which students learn through facilitated problem solving. Students work in small learning groups, guided by a facilitator, to solve a complex and realistic problem [7]. PBL is an educational approach where learners use scenarios or case studies, typically in small groups, to actively problem-solve and construct knowledge [8]. This student-cantered methodology emphasizes active participation, critical thinking, and the application of theoretical knowledge to practical situations. In PBL, learners identify their own knowledge gaps and learning needs, then work collaboratively to research and apply evidence to solve authentic problems [9].

Previous systematic reviews have examined PBL effectiveness in various healthcare education contexts [10] and teaching EBP to postgraduate medical students [11, 12]. However, no review to date has specifically assessed the efficacy of PBL as a teaching approach for EBP in nursing and midwifery education, representing a significant gap in the literature.

Therefore, this review aims to systematically evaluate the efficacy of Problem-Based Learning approaches in teaching Evidence-Based Practice to nursing and midwifery students and professionals.

Therefore, this review aims to systematically evaluate the efficacy of Problem-Based Learning approaches in teaching Evidence-Based Practice to nursing and midwifery students and professionals.

## Methods

### Protocol and registration

This systematic review was conducted according to the Cochrane Handbook for Systematic Reviews of Interventions [13] and reported following the PRISMA 2020 statement [14]. The review protocol was registered in PROSPERO (Registration number: CRD42023390989).

#### Clinical trial number

Not applicable. This study is a systematic review of published literature and does not constitute a clinical trial.

### Research question

The primary review question was: “What is the efficacy of Problem-Based Learning compared to other teaching approaches for developing Evidence-Based Practice competencies among nursing and midwifery students and professionals?”

### Eligibility criteria (PICO-Framework)

**Population:** Nursing and/or midwifery students at undergraduate or postgraduate levels, and practicing nurses and midwives

**Intervention:** Problem-Based Learning as a teaching approach for Evidence-Based Practice

**Comparison:** Other teaching methods (traditional lectures, case-based learning, simulation, etc.) or control groups

**Outcomes:** Evidence-Based Practice competencies measured by knowledge, attitudes, skills, or behaviours

Efficacy of PBL for teaching EBP was assessed through the following outcomes:

- **Knowledge outcomes:** Scores on validated instruments measuring EBP knowledge
- **Attitudinal outcomes:** Changes in attitudes toward EBP and confidence in applying EBP
- **Skill outcomes:** Critical appraisal skills, clinical decision-making abilities, and critical thinking scores
- **Behavioural outcomes:** Evidence-based practice implementation behaviours and self-reported competencies

Studies were included if they measured at least one of these outcomes using validated instruments or standardized assessments.

### Study design

We included qualitative, quantitative and mixed-methods studies with comparative designs, specifically: randomized controlled trials (RCTs), quasi-experimental studies with control/comparison groups, controlled before-and-after studies, and cohort studies with comparison groups. The inclusion of qualitative studies was justified to provide essential contextual understanding of implementation barriers, facilitators, and practitioner experiences that complement quantitative efficacy measures and explain how and under what conditions PBL interventions can be successfully implemented in real-world healthcare education settings. Single-group pre-post studies without controls were excluded to ensure rigor in assessing PBL efficacy.

### Inclusion criteria

- Peer-reviewed empirical studies published between 2001-2025
- Studies examining Problem-Based Learning as a teaching approach for EBP
- Participants: nursing and/or midwifery students and professionals
- Outcomes related to EBP competencies measured using validated instruments

### Exclusion criteria

- Non-peer reviewed publications
- Studies not specifically examining PBL for EBP teaching
- Studies focusing solely on other healthcare professions
- Opinion pieces, editorials, or narrative reviews without original data
- Single-group pre-post studies without comparison groups

### Information sources

A comprehensive literature search was conducted across multiple electronic databases from their inception to August, 2025. The databases searched included MEDLINE (via PubMed), CINAHL, EMBASE, Web of Science, ERIC, PsycINFO, Cochrane CENTRAL, and Google Scholar. In addition to the database search, the reference lists of all included studies were manually screened to identify any additional relevant articles that met the inclusion criteria.

### Search strategy

**Table:** 

(“Problem-Based Learning”[MeSH Terms] OR “Problem-Based Learning” OR “PBL“OR “Problem Based Learning”) AND (“Evidence-Based Practice”[MeSH Terms] OR “Evidence-Based Practice” OR “Evidence Based Practice” OR “EBP” OR “Evidence-Based Medicine”) AND (“Nursing Education”[MeSH Terms] OR “Midwifery”[MeSH Terms] OR “Nursing Students”[MeSH Terms] OR “Midwifery Students” OR “Nursing Education” OR “Midwifery Education” OR “Nursing Students” OR “Midwifery Students” OR “nurse“OR “midwife”)	

The search strategy was adapted for each database to account for different indexing systems.

### Study selection

Two reviewers (GK and AK) independently screened titles and abstracts using predetermined inclusion criteria. Full-text articles of potentially relevant studies were obtained and assessed for eligibility. Disagreements were resolved through discussion and consensus, with a third reviewer (OM) consulted for arbitration when required. Cohen’s kappa was calculated to assess inter-rater reliability for study selection (κ = 0.89, indicating excellent agreement). In line with established reporting standards, the study selection process was documented using the PRISMA 2020 flow diagram.

After database searches yielded 2,179 records from the initial search (conducted through August 2023) and an additional 252 records from the updated search (May 2024 - August 2025), all citations were imported into Covidence reference management software. The software’s automated deduplication function was used to identify exact and near-duplicate records based on title, authors, publication year, and DOI matching. This automated process removed 1,791 duplicates. Subsequently, two reviewers (GK and AK) manually screened the remaining records for any additional duplicates missed by the automated process, removing an additional 16 duplicates. This resulted in 388 unique records for title and abstract screening from the initial search and 236 records from the updated search, totalling 624 records for screening.

### Data collection process

Two reviewers (GK and AK) independently extracted data using a standardized data extraction form developed a priori and based on the Cochrane Collaboration’s data collection template. The extraction form was pilot-tested on a representative sample of included studies to ensure consistency and completeness prior to full implementation. Data extraction was performed in duplicate with reviewers working independently. Discrepancies between reviewers were systematically identified, discussed, and reconciled through consensus. A pre-specified protocol for involving a third reviewer to arbitrate unresolved disagreements was established, though consultation was not required.

### Data items

The extracted data included important characteristics of the studies, such as the first author, year of publication, country of origin, study design, and setting. The reviewers also recorded details about the participants, including sample size, age, gender distribution, and education level. Information about the intervention was documented, detailing the approaches to PBL, the duration of the interventions, and the comparison groups used. Additionally, the outcome measures and assessment tools employed in each study were systematically noted, along with the results and key findings. Furthermore, information needed for assessing the risk of bias was extracted to aid in the quality appraisal of the included studies.

### Risk of bias in individual studies

Risk of bias in the included studies was assessed independently by two reviewers (GK and OM) using the appropriate JBI-SUMARI tools according to the study design. Randomized controlled trials were appraised with the JBI Critical Appraisal Checklist for Randomized Controlled Trials (13 items), while quasi-experimental studies were evaluated using the JBI Critical Appraisal Checklist for Quasi-Experimental Studies (9 items). For cross-sectional studies, the JBI Critical Appraisal Checklist for Analytical Cross-Sectional Studies (8 items) was applied. In addition to these tools, the ROBINS-I instrument was used to assess risk of bias in non-randomized studies, focusing on seven domains: confounding, selection of participants, classification of interventions, deviations from intended interventions, missing data, measurement of outcomes, and selection of reported results. Any disagreements between the reviewers were resolved through discussion until consensus was reached.

### Software used

For the review process, Covidence (Veritas Health Innovation, Melbourne, Australia) was used to facilitate screening and data extraction, while Microsoft Excel 2021 supported data management. Statistical analyses and visualizations of risk of bias were conducted in R (version 4.3.0) using the ‘robvis’ package.

### Data synthesis and analysis

#### Narrative synthesis methodology

Given the heterogeneity in study designs, interventions, outcome measures, and populations, meta-analysis was not appropriate. Instead, we conducted a narrative synthesis following the guidance of Popay et al. (2006) [15] and the Cochrane Handbook for Systematic Reviews.

Our narrative synthesis involved four iterative stages:

##### Developing a preliminary synthesis

We organized the studies according to their focus, distinguishing between those that were PBL-specific and those that addressed broader EBP education. To facilitate comparison, we created summary tables that extracted and presented key study characteristics, interventions, outcomes, and findings.

##### Exploring relationships within and between studies

We examined patterns across the studies, identifying similarities and differences in PBL implementation approaches, educational contexts, outcome types and measurement strategies, as well as the reported directions and magnitudes of effects.

##### Assessing robustness of the synthesis

The robustness of our synthesis was assessed by considering the methodological quality of the included studies, the consistency of findings across different contexts, the potential influence of study design on outcomes, and the limitations or biases that may have shaped the evidence.

##### Developing themes

Through iterative reading and discussion within the review team, we identified six major themes related to PBL effectiveness, implementation, and existing evidence gaps. The synthesis was conducted independently by two reviewers (GK and VRL), with regular team discussions to ensure rigor and minimize interpretation bias. A meta-synthesis was not conducted, as the included studies were primarily quantitative.

#### Assessment of heterogeneity

We assessed heterogeneity across clinical, methodological, and statistical dimensions. Clinical heterogeneity reflected differences in populations (students vs. professionals, undergraduate vs. postgraduate, nursing vs. midwifery), while methodological heterogeneity arose from varied study designs, including randomized controlled trials, quasi-experimental, and cross-sectional studies. Statistical heterogeneity was not formally assessed, as meta-analysis was deemed inappropriate due to substantial variation in outcome measures. The decision not to conduct a meta-analysis was made a priori, and confirmed during data extraction, when marked differences emerged in PBL implementation models, outcome measurement instruments, intervention durations, and the availability of comparable control groups.

#### Summary measures and synthesis of results

Due to significant heterogeneity in study designs, interventions, and outcome measures, meta-analysis was not feasible. Therefore, narrative synthesis was conducted following established guidance [15]. The synthesis focused specifically on studies that examined PBL approaches for teaching EBP, with particular attention to effectiveness, implementation challenges, and factors influencing success.

## Results

### Study selection

The initial database searches conducted through August 2023 yielded 2,179 articles. Following the removal of 1,791 duplicates using Covidence software and manual verification, 388 unique articles underwent title and abstract screening.

During the title and abstract screening phase, 328 articles were excluded. Among these excluded articles, 108 were removed due to having an irrelevant study population that did not focus on nursing or midwifery students or professionals, while 142 articles examined interventions other than PBL or EBP teaching. Additionally, 56 articles did not measure EBP competencies as outcomes, and 22 articles had ineligible study designs such as reviews, protocols, or editorials. This screening process left 60 articles for full-text assessment.

During the full-text screening phase, 42 articles were subsequently excluded. The majority of these exclusions, comprising 38 articles, did not specifically examine PBL as a teaching approach for EBP. Three articles were excluded due to insufficient outcome data, and one article was excluded because the full text was unavailable. Following this rigorous screening process, eighteen studies met all inclusion criteria and were included in the qualitative synthesis.

The updated search conducted between May 2024 and August 2025 identified 236 additional records. After screening these records, 4 articles underwent full-text assessment but none met the inclusion criteria. Among these excluded articles, 2 had irrelevant outcomes, 1 had inappropriate methodology, and 1 had an incorrect study population.

**Fig. 1:**
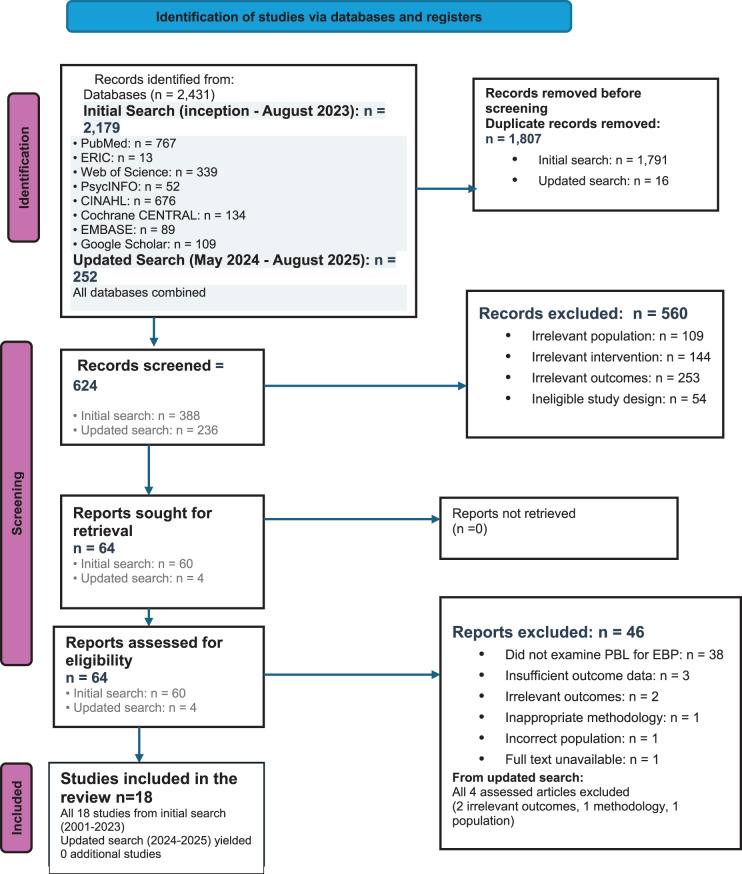
PRISMA flow diagram showing the complete study selection process with specific numbers at each stage. PRISMA flow diagram (Page et al., 2021)

Critically, only 3 studies, representing 16.7% of the included studies, specifically examined PBL as a teaching approach for EBP. The complete study selection process is illustrated in Fig. 1.

### Characteristics of included studies

The 18 included studies represented diverse geographical and economic contexts, with the United States contributing the largest number of studies (*n* = 4, 22.2%), followed by Ethiopia and Iran (*n* = 2 each, 11.1% each). Other countries including Australia, Germany, Israel, Japan, South Korea, Taiwan, and Iraq each contributed one study. Economic classification revealed 13 studies (72.2%) from high-income countries, 3 studies (16.7%) from middle-income countries, and 2 studies (11.1%) from low-income countries.

Professional focus analysis showed that 12 studies (66.7%) concentrated on nursing professionals, 4 studies (22.2%) included both nurses and midwives, and 2 studies (11.1%) focused specifically on midwifery practice. This distribution reflects the dominant representation of nursing research in healthcare education literature.

Methodologically, studies employed diverse approaches: cross-sectional designs were most common (*n* = 8, 38.1%), followed by mixed methods studies (*n* = 4, 19.1%), qualitative studies (*n* = 3, 14.3%), and equal numbers of randomized controlled trials and quasi-experimental studies (*n* = 2 each, 9.5%). Sample sizes varied considerably, ranging from 20 participants (Nango & Tanaka, 2010) to 3,397 participants (Harper et al., 2017). Response rates ranged from 7.4% to 100%, with several studies achieving full participation from their target populations, as shown in Table 1.

**Table 1:** Characteristics of included studies examining PBL and EBP education in nursing and midwifery (*N* = 18)

S/N	Author/Year	Country	Study design	Participants (Intervention and Control)	Study population	Sample size (n)	Level professional training (no’s)	Tools used	Variables measured	Main Results
1.	Adams (2009)	USA	Cross sectional	None	nurses	247	Bachelor (105) Post graduate (126)	Not indicated	Not specified	Found gaps in EBP knowledge and uptake
2.	Ahmed (2014)	Iraq	Cross sectional	None	midwives	53	Bachelor (53)	Not indicated	Not specified	Midwives had moderate EBP practice; barriers included lack of time & resources
3.	(Cheng, Huang, Yang, & Chang, 2020)	Taiwan	Quai-experimental	Experiential learning program vs. baseline	Nurses	103	1^st^ Yr. Bachelor students (103)	Not indicated	Not specified	Experiential program improved knowledge and confidence
4.	(Dagne & Beshah, 2021)	Ethiopia	Qualitative (FGDs, interviews, observation)	None	Nurses, midwives	86	Nur. BSc. (29) Post grad (3) BSc. Mid-41. Post. Grad (5)	Klein and Knight theory	Not specified	Challenges in EBP implementation related to organizational and resource barriers
5.	(Dagne, Beshah, Kassa, & Dagnaw, 2021)	Ethiopia	Cross sectional	None	Nurses and midwives	790	Not indicated	Not indicated	Asking clinical question, Evidence acquisition, Appraisal, Application, Evaluation	Poor implementation of EBP overall
6.	(Delaney, Apostolidis, Lachapelle, & Fortinsky, 2011)	USA	Cross sectional	None	Nurses	Not indicated	Not indicated	Not specified	Not specified	EBP knowledge was limited; highlighted need for education
7.	(Ehrenbrusthoff, Braun, Bahns, Happe, & Kopkow, 2022b)	Germany	Nationawide online survey	None	Midwives and nurses	889	Not indicated	Not specified	Not indicated	Midwives reported limited EBP knowledge and lack of training opportunities
8.	(Mashiach Eizenberg, 2011)	Israel	Cross sectional	**Intervention**: (EBP implementation) **Control:** (non- EBP implementers)	Nurses	243	Not indicated	Not specified	Not indicated	Found strong need for research-based information; gaps in EBP use
9.	(Fry & Attawet, 2018)	Australia	Online survey	None	Nurses and Midwives	204	Not indicated	Not specified	Not indicated	Four barriers identified: time, workload, access to resources, and confidence
10	(Gebresilassie, Baraki, Kassie, & Wami, 2022)	Ethiopia	Cross sectional	None	Midwives	314	Not indicated	Not specified	Not indicated	Research capacity of clinical midwives was low; recommended training
11	(Hauck, Winsett, & Kuric, 2013)	USA	Prospective descriptive comparative	None	Nurses	427	Not indicated	Rogers’s Diffusion of Innovations	Not indicated	EBP integration supported but uptake varied
12	(Jamshidi, Hemmati Maslakpak, & Parizad, 2021)	Iran	RCT	PBL-based education vs. routine lectures	Nurses	78	Not indicated	Not specified	Not indicated	PBL group had significantly higher EBP knowledge and skills
13	(Jamshidi, Parizad, & Maslakpak, 2021)	Iran	Quasi-experimental	PBL sessions vs. no session	Nurses	78	Not indicated	Not specified	Not indicated	PBL sessions improved knowledge and attitudes toward EBP
14	(Nango & Tanaka, 2010)	Japan	RCT	PBL learning vs. baseline	Nurses	20	Not indicated	Not specified	Not indicated	PBL improved problem-solving and critical thinking
16	(Son, 2020)	South Korea	Quasi-experimental	**Simulation PBL vs. traditional practicum**	Nurses	78	Not indicated	Not specified	Not indicated	Simulation PBL improved clinical reasoning and self-efficacy
17.	(Harper et al., 2017)	USA	Delphi survey	None	Nurses	3,397	75% Post grad. 25% Bachelor	Not specified	Not indicated	Experts agreed on competencies for EBP integration
18.	Gulzar MALIK (2016)	Australia	Qualitative grounded theory	None	Nurses and midwives	23	100% post grad	Purposive sampling, interview guide	Not indicated	Identified themes on barriers and enablers for EBP adoption

**Abbreviations:** RCT = Randomized Controlled Trial; PBL = Problem-Based Learning; EBP = Evidence-Based Practice; BSc = Bachelor of Science; Postgrad = Postgraduate; Nur = Nursing; BSc = Bachelor of Science; Mid = Midwife

### Risk of bias within studies

**Fig. 2:**
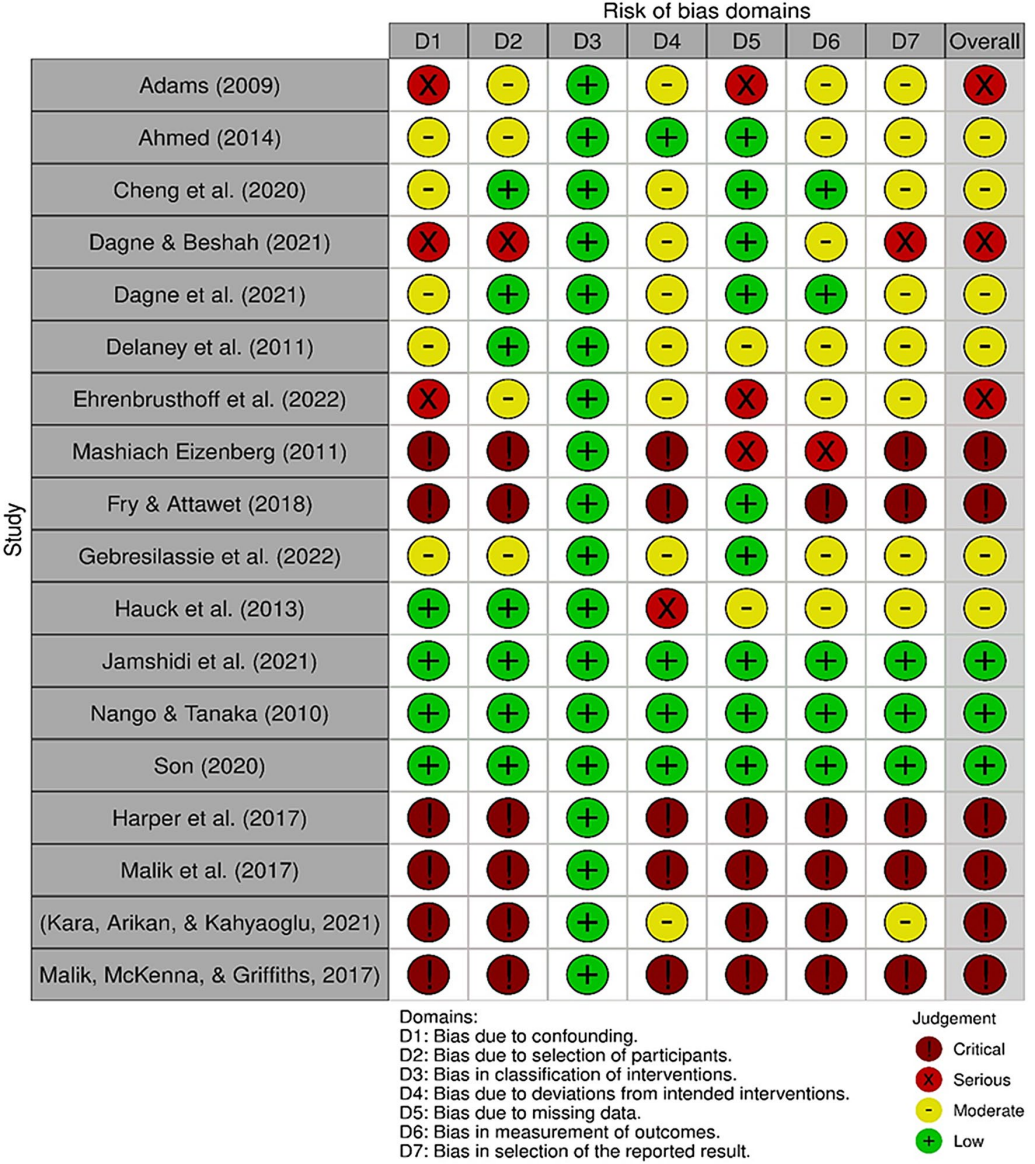
Risk of bias summary showing review authors’ judgements about each risk of bias item for each included study, presented as a traffic light plot. Green indicates low risk of bias, yellow indicates moderate risk, and red indicates serious risk of bias. Generated using the robvis R package (McGuinness LA, Higgins JPT. Risk-of-bias visualization (robvis): An R package and Shiny web app for visualizing risk-of-bias assessments. Res Syn Meth. 2020; 1–7. 10.1002/jrsm.1411)

Quality assessment using appropriate JBI tools revealed varying methodological rigor across the included studies. Three studies (16.7%) demonstrated low risk of bias across all domains, notably including all three PBL-specific studies (Jamshidi et al., 2021 [16]; Nango & Tanaka, 2010 [17]; Son, 2020 [18]), while six studies (33.3%) showed moderate bias, demonstrating acceptable methodological quality despite some limitations in areas such as confounding control or outcome measurement. However, three studies (16.7%) were categorized as having serious bias, indicating substantial methodological concerns including inadequate randomization, significant attrition, or unclear outcome reporting. A visual summary of the risk of bias assessment across all included studies is presented in Fig. 2, with the traffic light plot showing risk of bias assessment across all included studies using JBI and ROBINS-I tools.

### Summary of review findings

This review synthesized evidence from 18 studies comprising three randomized controlled trials, five quasi-experimental studies, eight cross-sectional surveys, and two qualitative studies (Table 2). Problem-based learning interventions demonstrated significant positive effects, with participants showing improvements in knowledge (MD: 4.23, *p* = 0.001), patient safety attitudes (MD: 3.76–5.12, *p* = 0.001), clinical decision-making (15.3-point improvement, *p* < 0.05), and critical thinking skills (MD: 0.76, *p* < 0.01). However, overall EBP implementation rates remained low at 34.7%, with nurses at 37.2% and midwives at 53.5%, despite 70% of practitioners holding positive attitudes toward EBP. Common barriers included limited time, insufficient resources, and heavy workloads, while facilitators encompassed higher education levels, organizational support, and access to library and internet resources. Knowledge assessments varied across clinical domains from 49.9% to 78.9%, with practitioners primarily relying on clinical practice guidelines, internet sources, and textbooks. The majority of studies demonstrated low to moderate risk of bias, supporting the reliability of these findings.

**Table 2:** Summary of the study findings (*N* = 18)

Author (Year)	Study design	Intervention Details	Sample Size	Outcomes Measured	Main Findings
Jamshidi et al. (2021)	RCT	Intervention: PBL sessions on patient safety and EBP (8 weeks). Control: Traditional lectures	78	Knowledge, attitudes, perceptions of patient safety	Significant improvements in intervention group: knowledge (MD: 4.23, *p* = 0.001), attitude (MD: 3.76, *p* = 0.001), patient safety perception (MD: 5.12, *p* = 0.001)
Nango & Tanaka (2010)	RCT	Intervention: Multidisciplinary PBL with medical students (4 weeks). Control: Standard conditions	20	Knowledge scores, clinical decision-making (VAS)	Significant improvement in clinical decision-making VAS in PBL group (mean improvement: 15.3 points, *p* < 0.05)
Son (2020)	Quasi-experimental	Intervention: Simulation-based PBL in maternity nursing practicum. Control: Traditional clinical practicum	78	Learning attitudes, critical thinking metacognition	Improvements in learning attitudes (MD: 0.89, *p* < 0.01) and critical thinking (MD: 0.76, *p* < 0.01)
Adams (2009)	Cross-sectional	Survey of EBP knowledge and information sources	247	EBP awareness, information sources, resource needs	87.3% aware via conferences, 87% via continuing education. Preferred sources: internet (65.5%), textbooks (48%). Identified need for resources, networking, guidelines
Ahmed (2014)	Cross-sectional	Survey of episiotomy practices	53	Clinical practice patterns, decision-making	Reasons for episiotomy: macrosomia (71.7%), breech (58.5%), shoulder dystocia (54.7%). Association between practice and experience/education level
Cheng et al. (2020)	Quasi-experimental	Experiential learning program vs. baseline	103	Critical thinking, learning behaviours	Significant pre-post improvements in behaviour, thoughts, feelings domains and in systematicity, open-mindedness, inquisitiveness, reflective thinking
Dagne & Beshah (2021)	Qualitative	Interview study on EBP implementation	86	EBP implementation experiences, barriers	Implementation rates: nurses 37.2%, midwives 53.5%. Identified barriers: time, resources, workload, awareness
Dagne et al. (2021)	Cross sectional	Survey of EBP implementation	790	EBP implementation rate, associated factors	34.7% implemented EBP moderately/desirably. Factors: age, attitude, nursing/midwifery work index, self-efficacy, knowledge
Delaney et al. (2011)	Cross-sectional	Survey of heart failure education knowledge	125	Knowledge level, educational needs	78.9% knowledge level on HF education principles. Requested information on HF topics, psychosocial issues, research evidence
Ehrenbrusthoff et al. (2022)	Cross sectional	Survey of EBP attitudes and behaviours	889	EBP attitudes, behaviours, implementation	70% positive attitude toward EBP. 80% self-rated able to enact EBP. < 70% preferred quantitative information over intuition. Implementation: nurses 13.4%, midwives 8.2%
Eizenberg (2011)	Cross sectional	Comparison of EBP implementers vs. non-implementers	243	Professional behaviour, resource access	Practitioners with degrees more evidence-based. EBP more likely with library access, computer access, internet searching opportunities
Fry & Attawet (2018)	Coss sectional	Survey of evidence sources	204	Evidence sources, searching frequency	Evidence primarily from clinical practice guidelines and publications. Statistical difference in searching frequency between leadership and other practitioners
Gebresilassie et al. (2022)	Cross sectional	Survey of research skills among midwives	314	Research conducting skills	54.8% (95% CI: 49.08%, 60.37%) had good research conducting skills
Hauck et al. (2013)	Cross sectional	Pre-post organizational intervention	427	Beliefs, organizational readiness	With interventions, total group scores for beliefs and organizational readiness to implement EBP improved significantly
Jamshidi et al. (2021b)	Quasi-experimental	PBL sessions on patient safety communication vs. no education	78	Patient safety communication skills	No significant difference in control group post-intervention (*p* = 0.162). Intervention group significantly higher scores after education (*p* = 0.001)
Kara et al. (2021)	Cross sectional	Survey of pressure injury prevention knowledge	108	Knowledge, attitudes, competence	Mean knowledge score 49.9%. Positive attitudes (mean 42.20 ± 2.40). Weak but significant positive correlation between knowledge and attitudes
Harper et al. (2017)	Delphi study	Expert consensus on educational priorities	3,397	Consensus on teaching priorities	Higher degree holders demonstrated more evidence-based professional behaviour. EBP
Malik et al. (2017)	Qualitative	Interview study on EBP teaching approaches	23	Teaching methods, challenges	Various pedagogical approaches used. Emphasis on literature searching and critical appraisal. Challenges: limited time, resources, workload, student disengagement

**Note:** PBL-specific studies are highlighted in bold. MD = Mean Difference; VAS = Visual Analog Scale; HF = Heart Failure; EBP = Evidence-Based Practice; PBL = Problem-Based Learning

## Results of individual studies

### PBL-specific studies for EBP education

#### Critical finding

Only 3 of the 18 included studies (16.7%) specifically examined PBL as a teaching approach for EBP among nursing students. No studies were identified that examined PBL for EBP education in midwifery specifically.

#### Jamshidi et al. (2021) - Iran [19]

This randomized controlled trial involved 78 nursing students randomly assigned to either PBL education (*n* = 39) or traditional lectures (*n* = 39). The PBL intervention consisted of structured problem-based learning sessions focused on patient safety and evidence-based practice over 8 weeks. Results demonstrated statistically significant improvements in the intervention group across multiple domains: knowledge scores (mean difference: 4.23, *p* = 0.001), attitude scores (mean difference: 3.76, *p* = 0.001), and perceptions of patient safety (mean difference: 5.12, *p* = 0.001) compared to the control group receiving routine lectures.

#### Nango & Tanaka (2010) - Japan [20]

This randomized controlled trial with 20 nursing students (10 intervention, 10 control) compared multidisciplinary PBL to standard baseline conditions. The intervention involved problem-based learning sessions with medical students over 4 weeks. While baseline knowledge scores showed no significant differences between groups, the Visual Analog Scale for clinical decision-making demonstrated significant improvement in the PBL group post-intervention (mean improvement: 15.3 points, *p* < 0.05), suggesting enhanced clinical reasoning abilities essential for EBP implementation.

#### Son (2020) - South Korea [21]

This quasi-experimental study involved 78 nursing students divided into experimental (*n* = 39) and control (*n* = 39) groups. The experimental group received simulation-based PBL (S-PBL) during maternity nursing clinical practicum, while the control group received traditional clinical practicum. Results showed the experimental group had significantly greater pre-post improvements in learning attitudes (mean difference: 0.89, *p* < 0.01) and critical thinking (mean difference: 0.76, *p* < 0.01) compared to controls. The study concluded that S-PBL was effective for improving nursing students’ learning transfer and metacognitive abilities.

### Non-PBL EBP education studies

The remaining 15 studies examined various approaches to teaching EBP but did not specifically focus on PBL methodology. These studies reported diverse EBP implementation rates: among nursing professionals, rates varied from 13.4% (Ehrenbrusthoff et al., 2022) to 100% (multiple studies), while among midwifery professionals, rates ranged from 34.6% (Dagne et al., 2021) to 100% (Gebresilassie et al., 2022). Common alternative teaching approaches included traditional lectures, blended learning, experiential learning, simulation-based methods, and clinical practicum projects. A comprehensive summary of study findings, including intervention details, outcomes measured, and key results for all included studies, is provided in Table 2.

### Summary of key findings

This systematic review identified a critical gap in the research literature. Of 18 studies meeting inclusion criteria after comprehensive searching, only 3 (16.7%) specifically examined PBL as a teaching approach for EBP education. These three studies, all conducted in Asian countries with nursing students, consistently demonstrated positive outcomes:

1. **Knowledge acquisition:** Significant improvements in EBP knowledge compared to traditional teaching (Jamshidi et al., 2021)
2. **Attitude development:** Enhanced attitudes toward EBP and patient safety across all three studies
3. **Critical thinking:** Significant improvements in critical thinking and analytical skills (Son, 2020)
4. **Clinical decision-making:** Enhanced clinical reasoning abilities (Nango & Tanaka, 2010)

All three PBL-specific studies demonstrated low risk of bias, lending credibility to their findings. However, the small number of studies, lack of midwifery-specific research, absence of long-term follow-up, and geographic concentration in Asia represent substantial limitations in the evidence base.

The broader EBP education literature (15 studies) revealed highly variable implementation rates and identified consistent facilitators (adequate resources, faculty preparation, organizational support) and barriers (time limitations, infrastructure constraints, faculty workload) to successful EBP education, regardless of teaching approach.

### Narrative synthesis of results

Following established guidance for narrative synthesis, the analysis was structured around key themes that emerged from the data, with particular focus on the three studies that directly addressed the research question.

### Theme 1: Effectiveness of PBL for developing EBP competencies

Knowledge acquisition: The three PBL-specific studies consistently demonstrated improvements in EBP-related knowledge. Jamshidi et al. (2021) reported the most robust evidence with significant knowledge improvements (*p* = 0.001) using validated instruments. This finding suggests that PBL's active learning approach effectively facilitates knowledge construction about EBP principles and processes.

#### Attitude development toward EBP

All three PBL studies reported positive attitudinal changes. Jamshidi et al. documented significant improvements in attitudes toward patient safety and EBP implementation. Son (2020) found enhanced learning attitudes, while Nango & Tanaka (2010) observed improved confidence in clinical decision-making. These attitudinal improvements are crucial since positive attitudes toward EBP strongly predict implementation behaviours in clinical practice.

#### Critical thinking and analytical skills

Two studies (Son, 2020; and one additional study by Cheng et al., 2020, which included PBL elements) demonstrated significant improvements in critical thinking dimensions including systematicity, analyticity, open-mindedness, and inquisitiveness. These cognitive skills are fundamental to the EBP process, particularly for critically appraising evidence and applying research findings to clinical decisions.

#### Clinical decision-making abilities

Nango & Tanaka (2010) provided specific evidence that PBL enhanced clinical decision-making skills, as measured by visual analogue scales. This finding is particularly relevant since clinical decision-making represents the ultimate application of EBP competencies in practice.

### Theme 2: Comparison of PBL with traditional teaching methods

The three PBL studies consistently demonstrated superiority over traditional lecture-based approaches. Jamshidi et al. (2021) showed significant advantages across knowledge, attitude, and perception domains when comparing PBL to routine lectures. Son (2020) found greater improvements in learning attitudes and critical thinking with S-PBL compared to traditional clinical practicum. This pattern suggests that active, problem-solving pedagogies may be more effective than passive learning approaches for developing complex EBP competencies.

### Theme 3: Implementation challenges and resource requirements

Analysis across all studies revealed consistent implementation challenges that must be addressed for successful PBL adoption:

#### Resource constraints

Multiple studies highlighted the need for adequate technological infrastructure, including computer access, internet connectivity, and database subscriptions. Mashiach Eizenberg (2011) specifically noted that EBP implementation was more likely where there was “access to a rich library with nursing and medical journals, and opportunities for working with a computer and for searching the Internet in the workplace.”

#### Time limitations

Studies consistently identified insufficient time as a barrier, both for conducting PBL sessions and for students to engage in evidence searching and critical appraisal activities. This challenge requires institutional commitment to curriculum redesign and adequate contact hours.

#### Faculty preparation

The need for instructor training in PBL facilitation emerged as a critical success factor. Faculty must develop skills in group facilitation, question formulation, and maintaining student engagement while avoiding the provision of direct answers that would undermine the problem-solving process.

#### Student adaptation

Initial resistance to active learning approaches was noted, particularly among students accustomed to passive, lecture-based learning. This requires careful orientation and ongoing support to help students adapt to increased responsibility for their learning.

### Theme 4: Factors influencing success

Educational level impact: Analysis of broader study findings revealed that higher educational qualifications consistently correlated with improved EBP behaviours. Studies by Mashiach Eizenberg (2011) and Harper et al. (2017) demonstrated that practitioners with higher degrees exhibited more evidence-based professional behaviour, suggesting that advanced education enhances capacity for EBP implementation.

#### Institutional support

Studies emphasized the importance of organizational factors including leadership support, allocated time for EBP activities, and opportunities for professional development. Hauck et al. (2013) specifically found that “leadership facilitation strategies” were essential for establishing evidence-based practice.

#### Small group learning environments

The PBL studies consistently utilized small group formats (typically 8–12 participants), which appeared to enhance peer learning, discussion quality, and individual engagement. This finding aligns with PBL pedagogical principles emphasizing collaborative learning.

### Theme 5: Methodological quality and outcome measurement

Measurement heterogeneity: A significant challenge identified was the diversity of outcome measures used across studies. The three PBL studies employed different instruments and assessment approaches, limiting the ability to synthesize quantitative findings or conduct meta-analysis. This heterogeneity reflects the broader challenge in EBP education research regarding standardized outcome measurement.

#### Follow-up limitations

None of the PBL studies examined long-term retention of knowledge or sustained changes in clinical practice behaviours. This represents a critical gap since the ultimate goal of EBP education is to influence actual practice patterns over time.

#### Cultural and contextual factors

The three PBL studies were conducted in different Asian countries (Iran, Japan, South Korea), raising questions about generalizability to other cultural and educational contexts. However, the consistency of positive findings across these diverse settings suggests potential broader applicability.

### Theme 6: Evidence gaps and research needs

Limited evidence base: The most striking finding was that only 3 of 18 studies specifically examined PBL for EBP education, representing a critical gap in the literature. This limitation severely constrains the ability to draw definitive conclusions about PBL effectiveness for this specific educational objective.

#### Absence of midwifery-specific research

No studies were identified that specifically examined PBL for EBP education in midwifery, despite midwifery being included in the search strategy. This represents a significant gap given the distinct educational and practice contexts of midwifery.

#### Lack of comparative effectiveness research

None of the studies directly compared PBL to other active learning approaches (such as case-based learning or simulation), limiting understanding of PBLs relative effectiveness compared to other student-cantered pedagogies.

This narrative synthesis reveals that while limited evidence suggests PBL may be effective for teaching EBP to nursing students, the evidence base is insufficient to support definitive conclusions or widespread implementation recommendations. The consistent positive findings across the three studies, despite their different contexts and methodologies, provide preliminary support for PBL's potential effectiveness, but highlight the urgent need for more rigorous, comparative research in this area.

## Discussion

This systematic review highlights a critical gap in the current evidence base regarding the effectiveness of problem-based learning (PBL) in teaching evidence-based practice (EBP) to nursing and midwifery professionals. Although our comprehensive search yielded thousands of initial records, only three studies directly addressed the research question. This scarcity of evidence has important implications for both educational practice and future research in healthcare education.

### Interpretation of the limited evidence base

The limited number of relevant studies is a key finding and requires cautious interpretation. Despite the well-recognized value of PBL in health professions education, the increasing emphasis on developing EBP competencies in nursing and midwifery curricula, and the strong theoretical alignment between PBL’s problem-solving processes and the evidence-seeking skills central to EBP, the research evidence remains sparse.

Several factors may account for this gap. First, many educational institutions may adopt PBL and integrate EBP training without conducting rigorous evaluations or disseminating findings in peer-reviewed literature. Second, isolating the specific effects of PBL from broader curricular reforms is methodologically complex, which may limit the feasibility of controlled studies. Third, because EBP training is often embedded across entire curricula rather than delivered as a distinct module, attributing outcomes specifically to PBL approaches becomes challenging. Finally, publication bias may favour studies with broader pedagogical or curricular foci, rather than those addressing individual teaching strategies such as PBL.

### What the three PBL studies reveal about effectiveness

Although the evidence base is limited, the three included studies provide valuable preliminary insights into the potential effectiveness of PBL for teaching EBP in nursing and midwifery education. All reported positive outcomes across several domains.

#### Knowledge and cognitive outcomes

Jamshidi et al. (2021) reported significant knowledge gains in an RCT, with a mean difference of 4.23 (*p* = 0.001). This finding suggests that the active learning strategies inherent in PBL effectively facilitate the acquisition of EBP principles. Such outcomes are consistent with constructivist learning theory, which emphasizes that learners construct understanding through active engagement with authentic problems.

#### Attitudinal development

All three studies found improvements in participants’ attitudes, including greater confidence in implementing EBP and more favourable perceptions of evidence-based approaches. These attitudinal changes are noteworthy, as positive attitudes toward EBP are a strong predictor of subsequent adoption and use in clinical practice.

#### Critical thinking and clinical reasoning

Son (2020) and Nango & Tanaka (2010) reported improvements in critical thinking and clinical reasoning, both of which are core competencies for EBP. These cognitive skills are essential for appraising evidence and applying research findings to inform clinical decisions.

The consistency of positive findings across different geographical contexts (Iran, Japan, and South Korea) and PBL variations (traditional PBL, multidisciplinary PBL, and simulation-based PBL) suggests a degree of robustness in outcomes. However, the small number of studies and the heterogeneity of interventions limit the strength of conclusions that can be drawn.

### Comparison with broader PBL and EBP education literature

Our findings are consistent with the broader PBL literature in healthcare education while extending understanding specifically into EBP education. Kong et al. [22] reported that PBL was superior to traditional approaches for developing critical thinking in nursing education (effect size = 0.36), although the study did not focus on EBP. Similarly, Shin and Kim’s meta-analysis [23] demonstrated moderate positive effects of PBL on critical thinking development. The current review shows more consistently positive outcomes, which may be explained by the close alignment between PBL methodology and EBP education. The processes of evidence seeking and critical appraisal that are central to EBP naturally align with the problem-solving orientation of PBL.

However, our findings diverge from some earlier meta-analyses that reported mixed results regarding PBL effectiveness. Vernon and Blake’s classic meta-analysis [17] found that PBL was superior for clinical knowledge acquisition but not for basic science knowledge. Dochy et al. [16] later reported small positive effects for PBL on knowledge and skills across disciplines, highlighting variability in effectiveness depending on outcomes and context. More recent nursing-specific reviews provide further evidence: Sharma et al. [24] found that PBL enhanced nursing students’ ability to analyse and evaluate clinical problems, while Wei et al. [18] reported moderate positive effects of PBL on critical thinking (SMD 0.47, 95% CI 0.33–0.61, *p* < 0.01) in a systematic review and meta-analysis.

The theoretical alignment between PBL and EBP is strong. Both emphasize active, student-cantered learning, application of knowledge to authentic problems, development of self-directed learning skills, critical thinking, integration of diverse information sources, and collaborative learning. This convergence suggests that PBL may be particularly well suited to EBP education. Nonetheless, further high-quality research is required to confirm this hypothesis and to identify moderating factors such as intervention design, facilitator role, learner characteristics, and institutional context.

### Implementation considerations

Analysis of the included studies revealed several key factors influencing the successful implementation of PBL for EBP education. Adequate resources, including technological infrastructure, reliable internet access, and access to library databases, were consistently identified as essential [25]. Time constraints emerged as a major barrier, underscoring the need for curriculum redesign and allocation of sufficient contact hours. Faculty development was also critical, as effective PBL facilitation requires skills in guiding group discussions, fostering inquiry, and maintaining student engagement [26].

Student adaptation represented another important consideration. Several studies reported initial resistance to PBL, highlighting the need for structured orientation and ongoing support to ease the transition from traditional didactic methods to more student cantered approaches [27]. Furthermore, higher educational levels were associated with stronger EBP behaviours [25, 28], suggesting that PBL may be particularly beneficial for advanced learners.

### Methodological considerations and limitations

Our review has several important limitations. The most substantial is the small evidence base, only three studies specifically addressed our research question, severely constraining our ability to draw definitive conclusions about PBL effectiveness for EBP education. The inability to conduct meta-analysis due to methodological heterogeneity prevented quantitative synthesis and effect size estimation across studies.

The lack of long-term follow-up data means we cannot assess sustained effects on clinical practice behaviors, the ultimate goal of EBP education. None of the PBL studies examined whether improved competencies translated into changed practice patterns over time.

Publication bias represents another potential limitation. Studies finding null or negative results for PBL effectiveness may be underrepresented in published literature, potentially leading to overly optimistic conclusions.

We cannot exclude the possibility that relevant studies were missed despite comprehensive searching, particularly if they used alternative terminology or were published in languages other than English. However, our multi-database search strategy, reference list screening, and updated searches minimize this risk.

Despite these limitations, our review has important strengths. We conducted comprehensive searches across eight databases using carefully constructed strategies, employed rigorous PRISMA methodology, used validated JBI tools for quality assessment, and involved multiple independent reviewers to minimize bias. The narrative synthesis approach was appropriate given study heterogeneity and allowed thematic exploration of implementation factors beyond simple effectiveness measures.

### Implications for practice

While preliminary evidence is promising, educational institutions should approach PBL implementation for EBP teaching with caution, ensuring the availability of adequate resources such as technological infrastructure, database access, and library services, as well as comprehensive faculty preparation through training in both PBL facilitation and EBP knowledge. Successful implementation also requires sufficient curriculum time for meaningful engagement, clear student orientation to the learning approach and expectations, and strong organizational support through leadership commitment and integration into the broader curriculum. Importantly, PBL should be viewed not as a standalone solution but as part of a comprehensive educational strategy, complemented by other evidence-based pedagogies and embedded within a wider institutional commitment to fostering an EBP culture.

### Implications for research

This review identifies critical gaps that future research should address. Rigorous randomized controlled trials are needed to compare PBL with other active learning approaches, supported by validated instruments to measure EBP competencies consistently. Studies should also examine long-term effects on clinical practice, include midwifery-specific contexts, and explore optimal implementation strategies across diverse educational and cultural settings. Comparative effectiveness and mechanism-focused research, ideally using theory-driven and mixed-methods designs, will be essential to clarify how PBL influences outcomes and to guide its effective integration into curricula.

## Conclusions

This systematic review identified a critical evidence gap regarding PBL effectiveness for teaching EBP to nursing and midwifery professionals. While only three studies specifically addressed this question, they consistently demonstrated positive outcomes, suggesting PBL may be an effective educational approach.

However, the limited evidence base is insufficient to support definitive recommendations for widespread PBL implementation in EBP education. The heterogeneity of interventions and outcome measures across the broader literature prevents clear conclusions about optimal teaching approaches.

Future research priorities include: urgent need for well-designed comparative studies examining PBL effectiveness for EBP education; use of randomized controlled designs with validated outcome measures; development of standardized instruments for measuring EBP competencies; research on optimal PBL implementation strategies and resource requirements; and studies examining sustained effects on clinical practice behaviours.

While current evidence suggests promise, educational institutions should approach PBL implementation for EBP teaching with careful planning, adequate resource allocation, and ongoing evaluation. This review underscores both the potential of PBL for EBP education and the critical need for high-quality research to establish its effectiveness definitively.

## Electronic supplementary material

Below is the link to the electronic supplementary material.

Supplementary Material 1

## Data Availability

All data generated or analysed during this study are included in this published article and its supplementary information files. The datasets used and/or analysed during the current study are available from the corresponding author on reasonable request.

## Abbreviations

EBP: Evidence-Based Practice
PBL: Problem-Based Learning
JBI: Joanna Briggs Institute
PRISMA: Preferred Reporting Items for Systematic Reviews and Meta-Analyses
RCT: Randomized Controlled Trial
PICO P: Population, Intervention, Comparison, Outcome
S-PBL: Simulation-based Problem-Based Learning
ROBINS-I: Risk of Bias in Non-randomized Studies of Interventions
