# Supplementary material for: Efficacy of Problem Based Learning approach for teaching Evidence Based Practice in midwifery and nursing education: a systematic review
Source: BMC Nurs. 2025 Nov 19;24:1412. doi: 10.1186/s12912-025-04101-w (PMC12629050; doi:10.1186/s12912-025-04101-w)
Supplement: Supplementary file 1 — Supplementary Material 1 [file 12912_2025_4101_MOESM1_ESM.docx]

**TITLE:** **Efficacy of Problem Based Learning approach for teaching Evidence Based Practice for midwifery and nursing professionals globally: a systematic review and a meta - analysis protocol**

**SEARCH STRATEGY**

**Databases:**

PUBMED, EMBASE, Web of Science, ERIC and Google Scholar

| **DATABASE SEARCHED/DATE** | **SEARCH STRATEGY** | **RESULTS** |
| --- | --- | --- |
|  |  |  |
| **PUBMED/6APR23**  **UPDATED 14 MAY 2024-UPDATED 30 JUNE 2025** |  |  |
| 1 | Nursing professional*[Text Word] OR  Midwifery professional*[Text Word] OR  nurse[Text Word] OR nurses[Text  Word] OR midwives[Text Word] OR  midwife[Text Word] OR ((nursing OR  midwifery) AND student*[Text Word])  OR Students, Nursing[Mesh] OR  Undergraduate nursing student*[Text  Word] OR graduate nursing  student*[Text Word] OR Nursing and  midwifery education[Text Word] OR  Nursing or midwifery training[Text  Word] | 1,126,557 |
| 2 | Baccalaureate[Text Word] OR Critical  appraisal[Text Word] OR  Knowledge[Text Word] OR Skill*[Text  Word] OR attitude*[Text Word] OR  self-efficacy[Text Word] OR  teaching[Text Word] OR Learning[Text  Word] OR education[Text Word] OR  midwifery education[Text Word] OR  medical education[Text Word] OR  Occupational therapy[Text Word] OR  Allied health[Text Word] OR Core  competences[Text Word] OR Clinical  practice[Text Word] OR Graduate  training[Text Word] OR Bachelors  training[Text Word] OR Undergraduate  training[Text Word] OR continuous  training[Text Word] OR training,  continuous[Text Word] OR nursing  curriculum[Text Word] OR midwifery  curriculum[Text Word] OR  Undergraduate nursing and midwifery  education[Text Word] OR Graduate  nursing and midwifery education[Text  Word] | 3,474,201 |
| 3 | 1 AND 2 | 425,385 |
| 4 | (problem-based leaning[Text Word] OR  problem based learning[Text Word] OR  problem-based learning[Mesh Terms]  OR problem-based curriculum[Text  Word] OR problem-based curricula[Text  Word] OR PBL[Text Word]) OR  collaborative learning[Text Word] OR  experiential learning[Text Word] OR  learner-centered instructional  approach[Text Word] OR self-directed  learning[Text Word] | 27,120 |
| 5 | (evidence-based practice[Text Word] OR  EBP[Text Word] OR Evidence-based  education[Text Word] OR Evidence-  based teaching[Text Word] OR  Evidence-based learning[Text Word]  OR evidence based[Text Word] OR  evidence-based nursing[Text Word]) | 251,607 |
| 6 | 4 OR 5 | 277,732 |
| 7 | 3 AND 6 | 24,147 |
| 8 | epidemiology[Text Word] OR prevalence[Text Word] OR incidence[Text Word] OR frequency[Text Word] OR etiology[Text Word] OR complications[Text Word] | 8,419,499 |
| 9 | 7 AND 8 | 4,307 |
| 10 | Cross-sectional[Text Word] OR observational study[Text Word] OR Case-control study[Text Word] OR Cohort study[Text Word] OR Quasi experimental study[Text Word] OR Time series[Text Word] OR Randomized controlled trial[Text Word] | 2,179,912 |
| 11 | 9 AND 10 | 767 |
|  | 2024/5/15 - 2025/6/30 | 109 |
|  |  |  |
| WEB OF SCIENCE – 6 APRIL 2023-UPDATED 15MAY-2024  **UPDATED 30 JUNE 2025**  1 | TI=(Nursing professional* OR Midwifery professional* OR nurse OR nurses OR midwives OR midwife OR ((nursing OR midwifery) AND student*) OR Undergraduate nursing student* OR graduate nursing student* OR Nursing and midwifery education OR Nursing or midwifery training) | 281,165 |
| 2 | TI=(Baccalaureate OR Critical appraisal OR Knowledge OR Skill* OR attitude* OR self-efficacy OR teaching OR learning OR education OR midwifery education OR medicine OR occupational therapy OR allied health OR core competences OR clinical practice OR training OR continuous training OR training, continuous OR medical education) | 5,730,223 |
| 3 | 1 AND 2 | 154,126 |
| 4 | ALL=(problem-based leaning OR problem based learning OR problem-based curriculum OR problem-based curricula OR PBL OR collaborative learning OR experiential learning OR learner-centered instructional approach OR self-directed learning) | 319,977 |
| 5 | ALL=  (evidence-based practice OR EBP OR Evidence-based education OR Evidence-based teaching OR Evidence-based learning OR evidence based OR evidence-based nursing) | 735,390 |
| 6 | 4 OR 5 | 1,044,673 |
| 7 | 3 AND 6 | 20,624 |
| 8 | ALL=(Epidemiology OR prevalence OR incidence OR frequency OR etiology OR complications) | 4,031,949 |
| 9 | 7 AND 8 | 3,274 |
| 10 | TI=  (Cross-sectional OR observational study OR Case-control study OR Cohort study OR Quasi experimental study OR Time series OR Randomized controlled trial) | 560,628 |
| 11 | 9 AND 10 | 339 |
|  | 2024/5/15 - 2025/6/30 | **93** |
|  |  |  |
| **EBSCOHOST (**ACADEMIC SEARCH PREMIER, ERIC)-15 May,2024  **UPDATED 30 JUNE 2025**  1 | ((("Nursing professional*" or "Midwifery professional*" or nurse or nurses or midwives or midwife or (("nursing or midwifery") and student*) or "undergraduate nursing student*" or "graduate nursing student*" or Nursing) and "midwifery education") or Nursing or "midwifery training").mp. | 12,537 |
| 2 | (Baccalaureate OR "Critical appraisal" OR Knowledge OR Skill* OR attitude* OR self-efficacy OR teaching OR learning OR education OR "midwifery education" OR medicine OR occupational therapy OR allied health" OR "core competenc*" OR "clinical practice" OR training OR "continuous training" OR training, continuous OR "medical education").mp. | 1,439,370 |
| 3 | 1 AND 2 | 10,534 |
| 4 | ("problem-based leaning" OR "problem-based learning" OR "problem-based curriculum" OR "problem-based curricula" OR PBL OR "collaborative learning" OR "experiential learning" OR "learner-centered instructional approach" OR "self-directed learning").mp. | 37,523 |
| 5 | ("evidence-based practice" OR EBP OR "evidence-based education" OR "evidence-based teaching" OR "evidence-based learning" OR "evidence based" OR "evidence-based nursing").mp. | 15,721 |
| 6 | 4 OR 5 | 52,917 |
| 7 | 3 AND 6 | 676 |
| 8 | (Epidemiology OR prevalence OR incidence OR frequency OR etiology OR complications).mp. | 52,454 |
| 9 | 7 AND 8 | 13 |
| 10 | (Cross-sectional OR observational study OR Case-control study OR Cohort study OR Quasi experimental study OR Time series OR Randomized controlled trial) | 16,832 |
| 11 | 9 AND 10 | 2 |
|  | 2023/4/5 - 2024/5/15 | 0 |
|  |  |  |
| GOOGLE SCHOLAR | (Midwifery professional* OR Nursing professional* OR graduate nursing and midwifery student*) AND (problem-based learning OR evidence-based practice) AND (Epidemiology OR prevalence OR incidence) AND (cross-sectional study OR cohort OR RCT OR Quasi) | 27,900 |
|  | 2023/4/5 - 2024/5/15 | 8,230  (exported the first 50) |
| TOTAL |  | 252 |
| DUPLICATES |  | 16 |
| BALANCE FOR SCREENING |  | 236 |
